# Artificial intelligence and the ongoing need for empathy, compassion and trust in healthcare

**DOI:** 10.2471/BLT.19.237198

**Published:** 2020-01-27

**Authors:** Angeliki Kerasidou

**Affiliations:** a The Ethox Centre, Nuffield Department of Population Health, University of Oxford, Old Road Campus, Oxford, OX3 7LF, England.

## Abstract

Empathy, compassion and trust are fundamental values of a patient-centred, relational model of health care. In recent years, the quest for greater efficiency in health care, including economic efficiency, has often resulted in the side-lining of these values, making it difficult for health-care professionals to incorporate them in practice. Artificial intelligence is increasingly being used in health care. This technology promises greater efficiency and more free time for health-care professionals to focus on the human side of care, including fostering trust relationships and engaging with patients with empathy and compassion. This article considers the vision of efficient, empathetic and trustworthy health care put forward by the proponents of artificial intelligence. The paper suggests that artificial intelligence has the potential to fundamentally alter the way in which empathy, compassion and trust are currently regarded and practised in health care. Moving forward, it is important to re-evaluate whether and how these values could be incorporated and practised within a health-care system where artificial intelligence is increasingly used. Most importantly, society needs to re-examine what kind of health care it ought to promote.

## Résumé

L'empathie, la compassion et la confiance sont des valeurs fondamentales d'un modèle de soins de santé centré sur les relations avec le patient. Mais ces dernières années, la quête d'efficacité dans le secteur, y compris au niveau économique, a souvent relégué ces valeurs au second plan et les professionnels de la santé ont donc eu du mal à les intégrer à leur pratique. De son côté, l'intelligence artificielle gagne en importance. Cette technologie devrait accroître l'efficacité tout en libérant du temps pour les professionnels de la santé, qui pourront ainsi se concentrer sur l'aspect humain des soins, notamment en établissant une relation de confiance et en faisant preuve d'empathie et de compassion envers les patients. Le présent article s'intéresse à l'idée d'un système de soins de santé efficace, qui repose sur l'empathie et la confiance, et à laquelle adhèrent les adeptes de l'intelligence artificielle. Il suggère que l'intelligence artificielle a le potentiel nécessaire pour transformer radicalement la manière dont l'empathie, la compassion et la confiance sont considérées et appliquées aujourd'hui dans le secteur de la santé. À l'avenir, il est essentiel de réexaminer l'importance de ces valeurs et la façon dont elles pourraient être incorporées et mises en œuvre dans un système de santé où l'intelligence artificielle devient peu à peu incontournable. Et surtout, la société a besoin de se demander quel modèle de soins de santé elle souhaite promouvoir.

## Resumen

La empatía, la compasión y la confianza son valores fundamentales de un modelo relacional de atención sanitaria centrado en el paciente. En los últimos años, la búsqueda de una mayor eficiencia en la atención sanitaria, incluida la eficiencia económica, ha dado lugar con frecuencia a que estos valores se vean relegados a un segundo plano, lo que dificulta que los profesionales sanitarios los incorporen en la práctica. La inteligencia artificial se utiliza cada vez más en la atención sanitaria. Esta tecnología promete una mayor eficiencia y más tiempo libre para que los profesionales sanitarios se centren en el lado humano de la atención, lo que incluye el fomento de las relaciones de confianza y el trato a los pacientes con empatía y compasión. En este artículo se examina la visión de una atención sanitaria eficiente, empática y confiable que proponen los defensores de la inteligencia artificial. El artículo sugiere que la inteligencia artificial tiene el potencial de alterar fundamentalmente la forma en que la empatía, la compasión y la confianza se consideran y practican actualmente en la atención sanitaria. Para avanzar, es importante volver a evaluar si dichos valores se podrían incorporar y practicar en un sistema de atención sanitaria en el que se utiliza cada vez más la inteligencia artificial, y de qué manera. Lo más importante es que la sociedad debe reconsiderar qué tipo de atención sanitaria debe promover.

## ملخص

التعاطف والشفقة والثقة هي القيم الأساسية لنموذج الرعاية الصحية المرتكزة على المريض. في السنوات الأخيرة، أدى السعي لتحقيق المزيد من الفعالية في الرعاية الصحية، بما في ذلك الفعالية الاقتصادية، في الغالب إلى تباعد هذه القيم، مما جعل من الصعب على أخصائيي الرعاية الصحية دمجها في الممارسة. يُستخدم الذكاء الاصطناعي بشكل متزايد في الرعاية الصحية. وتقدم هذه التكنولوجيا وعوداً بمزيد من الفعالية والوقت الحر لأخصائيي الرعاية الصحية للتركيز على الجانب الإنساني من الرعاية، بما في ذلك تعزيز علاقات الثقة والاندماج مع المرضى من خلال التعاطف والشفقة. يناقش هذا المقال رؤية تتميز بالفعالية والتعاطف لرعاية صحية جديرة بالثقة، يطرحها مؤيدي الذكاء الاصطناعي. تشير الورقة إلى أن الذكاء الاصطناعي لديه إمكانية تغيير الطريقة التي ينظر بها إلى التعاطف والشفقة والثقة، وكيفية ممارسة كل منها، بشكل جذري في مجال الرعاية الصحية. ومع المضي قدما، من الهام إعادة تقييم ما إذا كان يمكن دمج وممارسة هذه القيم، داخل نظام الرعاية الصحية، حيث يستخدم الذكاء الاصطناعي بشكل متزايد، وكيفية القيام بذلك. والأهم من ذلك، يحتاج المجتمع إلى التحقق من نوع الرعاية الصحية الذي يمكن لهذه القيم أن ترتقي به.

## 摘要

理解、同情和信任是以患者为中心的、关系型医疗保健模式的基本价值。近年来，为了提高医疗保健的效率，包括经济效率，往往导致对这些价值观的背离，使医疗保健专业人员难以将其纳入实践。人工智能正越来越多地被应用于医疗保健。这项技术为医疗保健专业人员提供了更高的效率和更多的空闲时间，使他们能够专注于人性化的护理，包括培养信任关系，理解并同情患者。本文探讨了人工智能倡导者提出的高效、理解、可信赖的医疗保健理念。本文表明，人工智能有可能从根本上改变目前人们在医疗保健中看待和实践理解、同情和信任的方式。展望未来，重新评估这些价值观是否以及如何能够在越来越多地使用人工智能的医疗保健系统中纳入和实施是一件十分重要的事。最重要的是，社会需要重新审视什么类型的医疗保健值得推广。

## Резюме

Эмпатия, сочувствие и доверие — это основополагающие ценности ориентированной на пациента реляционной модели здравоохранения. В последнее время стремление повысить эффективность систем здравоохранения, в том числе их рентабельность, приводит к тому, что этим ценностям часто не уделяется должного внимания, что в свою очередь значительно осложняет их использование на практике работниками сферы здравоохранения. Применение искусственного интеллекта в сфере здравоохранения неуклонно растет. Эта технология привлекательна перспективой повышенной эффективности и тем, что она оставляет медицинским работникам больше свободного времени для непосредственной работы с пациентами, в том числе для налаживания доверительных отношений и применения эмпатии и сочувствия в профессиональном общении с пациентами. В этой статье рассматривается представление об эффективной системе здравоохранения, построенной на основе эмпатии и доверия, которое предлагается специалистами, продвигающими внедрение технологий ИИ в сфере здравоохранения. В статье выдвигается предположение о том, что искусственный интеллект потенциально способен коренным образом изменить сегодняшнее представление о применении эмпатии, сочувствия и доверия в сфере здравоохранения и внедрении соответствующих практик. В дальнейшем важно заново оценить возможность включения этих ценностей в систему здравоохранения, все чаще использующую технологию искусственного интеллекта, и их применения на практике. Что наиболее важно, общество нуждается в пересмотре того, развитие какого типа системы здравоохранения следует поощрять.

## Introduction

Empathy, compassion and trust are fundamental values of a patient-centred, relational model of health care. In recent years, the pursuit of greater efficiency in health care, including economic efficiency, has often resulted in these values being side-lined, making it difficult or even impossible for health-care professionals to incorporate them in practice. Artificial intelligence is increasingly being used in health care and promises greater efficiency, and effectiveness and a level of personalization not possible before. Artificial intelligence could help improve diagnosis and treatment accuracy, streamline workflow processes, and speed up the operation of clinics and hospital departments. The hope is that by improving efficiency, time will be freed for health-care professionals to focus more fully on the human side of care, which involves fostering trust relationships and engaging with patients, with empathy and compassion. However, the transformative force of artificial intelligence has the potential to disrupt the relationship between health-care professionals and patients as it is currently understood, and challenge both the role and nature of empathy, compassion and trust in this context. In a time of increasing use of artificial intelligence in health care, it is important to re-evaluate whether and how these values could be incorporated and exercised, but most importantly, society needs to re-examine what kind of health care it ought to promote. 

## Empathy, compassion and trust

Over the past decades, the rise of patient-centred care has shifted the culture of clinical medicine away from paternalism, in which the therapeutic relationship, the relationship between the health-care professional and the patient, is led by medical expertise, towards a more active engagement of patients in shared medical decision-making. This model of engagement requires the health-care professional to understand the patient’s perspective and guide the patient in making the right decision; a decision which reflects the patient’s needs, desires and ideals, and also promotes health-related values.^1^ The central point of the patient-centred model of doctor–patient relationship is that medical competency should not be reduced to technical expertise, but must include relational moral competency, particularly empathy, compassion and trust.^2^

Empathy, compassion and trust are broadly recognized as fundamental values of good health-care practice.^3^^–^^5^ Empathy allows health-care professionals to understand and share the patient’s feelings and perspective.^6^ Compassion is the desire to help, instigated by the empathetic engagement with the patient.^7^^,^^8^ Patients seek out and prefer to engage with health professionals who are competent, but also have the right interpersonal and emotional skills. The belief and confidence in the professional’s competency, understanding and desire to help is what underpins patient trust.^9^^–^^13^ Research has demonstrated the benefits of patient trust and empathetic care, including improved patient satisfaction, increased treatment adherence and improved health outcomes.^14^^,^^15^

Despite their importance, empathy and compassion in health care are often side-lined. In recent years, for example, socioeconomic factors, including an ageing population and austerity policies in Europe that followed the 2008 economic collapse, have led to the marginalization of these values.^16^ As health-care systems struggle with resourcing, the space for empathy and compassion has shrunk while the need for efficiency has grown.^17^ In the United Kingdom of Great Britain and Northern Ireland, high-profile cases and reports, such as the Francis report, which followed the Mid Staffordshire scandal,^18^ the report by the Health Service Ombudsman entitled *Dying without dignity,*^19^ and the Leadership Alliance for the Care of Dying People report,^20^ all pointed at the lack of empathy as a major problem in clinical care. What these cases also showed was a conflicting relationship between the need for empathy and the pursuit of greater economic efficiency and of meeting operational targets. In 2017, Sir Robert Francis, who chaired the inquiry into the Mid Staffordshire scandal, mentioned in an interview that “at the time at Mid Staffordshire there was huge pressure on organizations to balance their books, to make productivity improvements and matters of that nature. It all became about figures in the books, rather than outcomes for the patient. And I do believe there’s a danger of that happening again.”^21^ Research in 2017 in accident and emergency departments in England on the effect of austerity policies on the everyday experiences of health-care professionals found that the pressure to meet targets negatively affected the doctors’ and nurses’ ability and opportunity to practise empathetic and holistic care,^22^ which led to moral distress and burnout among these professionals.^23^

Against this backdrop, artificial intelligence has been heralded as a way to save struggling national health-care systems^24^ and transform the future of health care by providing greater efficiency, effectiveness and high levels of personalized care.^25^

## Artificial intelligence in health care

Artificial intelligence is broadly defined as “computing technologies that resemble processes associated with human intelligence, such as reasoning, learning and adaptation, sensory understanding, and interaction.”^26^ The hope is that these technologies will transform health-care delivery by“ streamlining workflow processes […] improving the accuracy of diagnosis and personalizing treatment, as well as helping staff work more efficiently and effectively.”^25^ Artificial intelligence could help health-care systems achieve greater efficiency, including economic efficiency, in two ways: (i) by improving time to and accuracy of diagnosis and treatment for patients, and where possible assisting with early prevention; and, (ii) by using health-care staff more efficiently.

A report published in 2018 in the United Kingdom suggested that the national health system could save up to 10% of its running costs by outsourcing repetitive and administrative tasks to artificial intelligence technologies.^24^ The same report also envisaged bedside robots performing social-care tasks such helping patients to eat, wash and dress, thus reducing the workload on care staff by 30%. But it is not only nursing and administrative tasks that artificial intelligence can help with. With regard to effectiveness, artificial intelligence systems could be used to deliver better clinical services both by assisting with the diagnosis and management of patients, and by providing the diagnosis and prescribing treatments. Research conducted so far has shown that machines can perform as well as, or even better than, humans in detecting skin cancer,^27^ heart arrhythmia^28^ and Alzheimer disease.^29^ Furthermore, human–machine partnerships can provide far better results than either humans or machines alone.^30^ In these examples, the principal benefits of artificial intelligence stem from its ability to improve efficiency and effectiveness by guiding diagnoses, delivering more accurate results and thus eliminating human error. With regard to greater efficiency through prevention, artificial intelligence technologies that track and analyse the movement of individuals could be used to detect people at risk of stroke and eliminate that risk through early intervention.^31^

Health care is already using technology to improve its efficiency and effectiveness. From scalpels and syringes to stethoscopes and X-ray machines, the list of technologies used in medicine to facilitate and improve patient care is long. However, artificial intelligence differs from previous medical technological advances. Whereas previous technologies were used to increase the senses and physical capacities of health-care professionals, consider, for example, how the stethoscope enhanced the hearing of doctors and X-rays their vision, the main role of artificial intelligence is to increase their reasoning and decision-making capacities. In this way, artificial intelligence is entering the health-care arena as another morally relevant actor that assists, guides or makes independent decisions regarding the treatment and management of patients.

Proponents of artificial intelligence technology in health care maintain that outsourcing tasks and decisions to rational machines will free up time for health-care professionals to engage in empathetic care and foster trust relationships with patients.^4^^,^^25^^,^^32^^,^^33^ A review, outlining recommendations for National Health Service to be the world leader in using technology to benefit patients, notes that while artificial intelligence cannot deliver indispensable human skills, such as compassion and empathy, “the gift of time delivered by the introduction of these technologies […] will bring a new emphasis on the nurturing of the precious inter-human bond, based on trust, clinical presence, empathy and communication.”^25^

The hope is that more free time for health-care professionals would not only lead to more trustworthy and empathetic care for patients, but also to less stress for and burnout of doctors and nurses.^34^ In addition, despite concerns that artificial intelligence will lead to job losses in health care, a report by the British Academy on the impact of artificial intelligence on work pointed out that professions that require the application of expertise and interaction with people will be less affected by automation through artificial intelligence.^35^ According to these aforementioned publications, the introduction of artificial intelligence technologies in health care offers the possibility of a win–win situation: patients benefit from more accurate diagnosis, better treatment outcomes, and increased empathy and compassion from medical staff, who in turn experience greater job satisfaction and less burnout.

The reimagination of health care, where artificial intelligence takes over specific, and even specialist, tasks while freeing time for health-care professionals to communicate and empathize with patients, assumes that the value attached to empathy, compassion and trust will remain high. However, patients and the health-care system might value accuracy and efficiency more than empathy and judgement, which could shift the focus in medicine away from human-specific skills.^36^ In which direction health-care delivery will evolve is an important theoretical and practical question that requires examination. Currently, it is still unclear whether and how health-care practice will be transformed by artificial intelligence, and what effect it may have, particularly on the role of health-care professionals and on the therapeutic relationship.

## Potential implications of artificial intelligence

Clinical competency is a fundamental aspect of the identity of health-care professionals and underpins the trust relationship between doctors and patients. Patient trust is based on the belief that doctors and nurses have the right skills and expertise required to help the patient and also the right motivation to do so. This combination of clinical skill with empathy and compassion is what justifies patients assuming a position of vulnerability towards the health-care professionals. Vulnerability is a fundamental characteristic of a trust relationship.^37^ The person placing trust in another knows and accepts that this trusted person can decisively influence the outcome of the entrusted action. Trust relationships involve a degree of uncertainty that cannot be mitigated; it is only the belief in the trusted person’s abilities and good will that justifies taking on the risk of this uncertainty. In the clinical context, the patient knows that things can go wrong, but believes and hopes that this wrong would not be intentional, but rather because of bad luck or unforeseeable circumstances. Rules and regulations are put in place to protect patients from negligence and preventable mistakes. The constant quest to improve care highlights the fundamental moral obligations of non-maleficence and of acting in the best interests of patients. However, the fact remains that, in some cases, preventable harm could be the outcome of a medical action.

The use of artificial intelligence to optimize accuracy of diagnosis and treatment could raise issues of accountability when things go wrong, not only in cases where doctors follow the recommendations of artificial intelligence, but also when they decide to override these recommendations.^38^ In such situations, it is unclear who should be held accountable, whether responsibility should lie with the algorithm developer, the data provider, the health system that adopted the artificial intelligence tool, or the health-care professional who used it. In addition, even in situations where the role of artificial intelligence is assistive, health-care professionals might not feel confident to override its recommendation. If machines are brought into health care because they are better than humans at making certain rational decisions, how could humans rationally argue against them? Yet, the question of accountability is not the only issue raised here. The role and nature of trust in the therapeutic relationship is also at stake. Would and should patients still trust health-care professionals? If the introduction of artificial intelligence tools results in outsourcing clinical and technical skills to machines, would a belief in the good will of the doctor be enough to sustain a therapeutic trust relationship as currently understood? One of the great promises of artificial intelligence is that by increasing effectiveness, accuracy and levels of personalization in clinical care, it will succeed in replacing trust with certainty.^39^ In this case, patients might stop considering health-care professionals as experts in whose skills and knowledge they need to trust. This change might lead to a different relationship between health-care professionals and patients, one not characterized by vulnerability, but one of an assistive partnership.^2^ However, even in this more positive scenario, the transformation of society’s expectations of care provision and the role of health-care professionals are unclear. It is important therefore to consider how the introduction of artificial intelligence will alter the public’s perception and understanding of trust in the clinical encounter as well as the way in which trust relationships will be formed in this context.

Similarly, artificial intelligence calls into question the role and value of empathy and compassion in health care. As mentioned earlier, in patient-centred care, empathy allows health-care professionals to understand the patients’ perspective, and thus helps health professionals tailor care to promote the patients’ values and address their individual needs. Empathy and compassion therefore play a very important role in an interpersonal model of care that rejects medical paternalism and brings the doctor and the patient together to discuss options and find appropriate solutions.^40^ To preserve this ideal of patient-centred care, artificial intelligence systems should be built in a way that allows for value-plurality, meaning the possibility that different patients might hold different values and have different priorities related to their care.^41^ In this way, the ethical ideal of shared decision-making can be maintained and not be replaced by another form of paternalism, one practised not by doctors, but by artificial intelligence algorithms.

Even if artificial intelligence tools are able to operate in a care context characterized by value-plurality, the role of empathy remains unclear. If what patient-centred care needs to survive in a future of artificial intelligence health care is machines programmed to incorporate more than one value, what does this mean about the nature and role of empathy in care provision? Is empathy still a professional value, or should it be now understood as another technology to be written into code and optimized? Indeed, research in the field of artificial intelligence suggests that it is possible to create empathetic machines^42^^,^^43^ as a way of relieving doctors and nurses from the substantial emotional work their professions require.^44^ The likely effects of such complete optimization and operationalization of health care are unclear. This optimization could improve health-care outcomes and personalized care; alternatively, it could lead to the reinstitution of a reductionist approach to medicine.^45^^,^^46^ Beyond these practical concerns, one should also consider whether something intangible, yet morally important will be lost if the therapeutic relationship is reduced to a set of functions performed by a machine, however intelligent. On the other hand, will our current understanding of empathy, compassion and trust change to fit the new context where some parts of care are provided by intelligent machines?

## Conclusion

The potential impact of artificial intelligence on health care, in general, and on the therapeutic relationship between health-care providers and patients, in particular, is widely acknowledged,^38^^,^^47^^,^^48^ as is the fact that society needs to learn how to deal “with new forms of agents, patients and environments.”^49^ Artificial intelligence has great potential to improve efficiency and effectiveness in health care. However, whether artificial intelligence can support other values central to the delivery of a patient-centred care, such as empathy, compassion and trust, requires careful examination. Moving forward, and as artificial intelligence is increasingly entering health care, it is important to consider whether these values should be incorporated and promoted within the new type of health care that is emerging and, if yes, how. More importantly, it is crucial to reflect on what kind of health care society should promote and how new technologies, including artificial intelligence, could help achieve it.
